# Turbulence Model Selection for Low Reynolds Number Flows

**DOI:** 10.1371/journal.pone.0153755

**Published:** 2016-04-22

**Authors:** S. M. A. Aftab, A. S. Mohd Rafie, N. A. Razak, K. A. Ahmad

**Affiliations:** 1 Dept of Aerospace Engineering, Universiti Putra Malaysia, Selangor, 43400, Malaysia; 2 School of Aerospace Engineering, Engineering Campus, Universiti Sains Malaysia, Nibong Tebal, 14300, Pulau Pinang, Malaysia; North China Electric Power University, CHINA

## Abstract

One of the major flow phenomena associated with low Reynolds number flow is the formation of separation bubbles on an airfoil’s surface. NACA4415 airfoil is commonly used in wind turbines and UAV applications. The stall characteristics are gradual compared to thin airfoils. The primary criterion set for this work is the capture of laminar separation bubble. Flow is simulated for a Reynolds number of 120,000. The numerical analysis carried out shows the advantages and disadvantages of a few turbulence models. The turbulence models tested were: one equation Spallart Allmars (S-A), two equation SST K-*ω*, three equation Intermittency (*γ*) SST, k-kl-*ω* and finally, the four equation transition *γ-Re*_*θ*_ SST. However, the variation in flow physics differs between these turbulence models. Procedure to establish the accuracy of the simulation, in accord with previous experimental results, has been discussed in detail.

## 1 Introduction

Low Reynolds number flow pose a great challenge in the selection of a Turbulence model for simulation. Many of the UAV’s and MAV’s work in these Reynolds number range. Colossal interest is growing in the CFD study of static wing and flapping wing aerodynamics in this regime [1].

In the case of low Re airfoils, the resistance to separation of the boundary layer is very poor, thus resulting in a dominant adverse pressure gradient. As flow separates from the point of minimum pressure, due to the increase in adverse pressure at the leading edge, separation takes place. The separated flow is highly unstable, resulting in transition immediately downstream, causing the flow to become turbulent. Thereby turbulent shear stresses energise the flow to counteract the increased adverse pressure, helping the flow to reattach. Thus, a zone in between separation and reattachment is formed, known as the separation bubble Mueller et al., [2] and Carmichael [3]. The separation bubble is dependent on the flow Re, the pressure distribution, the curvature of the airfoil, roughness and various other factors Gad-el-hak [1]. Two types of separation bubble exist, namely the short bubble and the long bubble. A short bubble exists when the flow Re is below 10^5^ and only extends to a couple of percent along the chord. The stability of this bubble is only for a short duration. Carmichael [3] has stated that below Re 5 × 10^4^, a laminar separation bubble causes a drastic drop in lift. If the Reynolds number exceeds 10^5^, a long bubble is formed. This bubble extends to 20–30% along the chord and affects the flow drastically [4]. For airfoils operating in the Re range of 10^6^, the adverse pressure gradient is eliminated by turbulent flow at transition thus preventing separation. An increase in Re induces turbulence in the boundary layer, imparting high energy to oppose separation.

One single turbulence model cannot be used as an ultimate solution for all simulations. Currently many commercial codes have incorporated new turbulence models to accurately model the flow behavior in the transition regime. Previously used turbulence models are tweaked or new models are developed, to accommodate the effect of transition on aerodynamic behaviour. Choudary et al, [5] have recently conducted a study on a NACA0021 airfoil using two transition models (k-kl-*ω* and transition *γ-Re*_*θ*_ SST) and have reported that k-kl-*ω* is more reliable for predicting separation bubble formation, growth and reattachment for their case.

The aim of the current work is to determine the separation bubble characteristics. A numerical analysis has been carried out using five turbulence models: the one equation S-A, two equation SST K-*ω*, three equation Intermittency(*γ*) SST, k-kl-*ω* and four equation *γ-Re*_*θ*_ SST turbulence model. The results of the simulation at Reynolds number 120,000 are compared with the experimental work carried out by Karthikeyan et al.[6].

## 2 Turbulence Modeling

### 2.1 Reynolds Average Navier Stoke(RANS)

In CFD, RANS is the most widely used turbulence modelling approach. In this approach, the Navier Stokes equations are split into mean and fluctuating components. The total velocity *u*_*i*_ is a function of the mean velocity ${\bar{u}}_{i}$ and the fluctuating velocity ${\overset{´}{u}}_{i}$ as shown in the equation below.

$$\begin{array}{c} u_{i}={\bar{u}}_{i}+\overset{´}{u_{i}} \end{array}$$

The continuity and momentum equation incorporating these instantaneous flow variables are given by

$$\begin{array}{c} \frac{\partial \rho }{\partial t}+\frac{\partial }{\partial X_{i}}\left(\rho u_{i}\right)=0 \end{array}$$

$$\begin{array}{c} \frac{\partial }{\partial t}\left(\rho u_{i}\right)+\frac{\partial }{\partial x_{i}}\left(\rho u_{i}u_{j}\right)=\frac{\partial \rho }{\partial x_{i}}+\frac{\partial }{\partial x_{j}}\left[\mu \left(\frac{\partial u_{i}}{\partial x_{j}}+\frac{\partial u_{j}}{\partial x_{i}}-\frac{2}{3}\delta_{ij}\frac{\partial u_{i}}{\partial x_{i}}\right)\right]+\frac{\partial }{\partial x_{i}}\left(-\rho \bar{\overset{´}{u_{i}\overset{´}{u_{i}}}}\right) \end{array}$$

These above equations (in Cartesian tensor form) are known as RANS equations, and the additional Reynolds stress terms $-\rho \bar{\overset{´}{u_{i}\overset{´}{u_{j}}}}$ need to be modelled. The Boussinesq hypothesis is applied in relating the Reynolds stress and mean velocity:

$$\begin{array}{c} -\rho \bar{\overset{´}{u_{i}\overset{´}{u_{j}}}}=\mu_{t}\left(\frac{\partial u_{i}}{\partial x_{j}}+\frac{\partial u_{j}}{\partial x_{i}}\right)-\frac{2}{3}\left(\rho k+\mu_{t}\frac{\partial u_{k}}{\partial x_{k}}\right)\delta_{ij} \end{array}$$

### 2.2 Spallart Allmars

The S-A turbulence model is a one-equation model, designed for aerospace applications. It is quite robust and effective in modelling the flow on an airfoil, with adverse pressure gradients in the boundary layer [7, 8].

The modified continuity equation for S-A solves the turbulent viscosity $\tilde{v}$.

$$\begin{array}{c} \frac{\partial }{\partial t}\left(\rho \tilde{v}\right)+\frac{\partial }{\partial x_{i}}\left(\rho \tilde{v}u_{i}\right)=G_{v}=\frac{1}{\sigma_{\tilde{v}}}\left[\frac{\partial }{\partial x_{j}}\left\{\left(\mu +\rho \tilde{v}\right)\frac{\partial \tilde{v}}{\partial x_{j}}\right\}+C_{b2\rho }{\left(\frac{\partial \tilde{v}}{\partial x_{j}}\right)}^{2}\right]-Y_{v}+S\tilde{v} \end{array}$$

*G*_*v*_ is the production of turbulent viscosity and *Y*_*v*_ is the destruction of turbulent viscosity.

The turbulent viscosity is calculated as shown

$$\begin{array}{c} \mu_{t}=\rho \tilde{v}f_{v1} \end{array}$$

The *f*_*v*1_ is the viscous damping function

$$\begin{array}{c} f_{v1}=\frac{\chi^{3}}{\chi^{3}+C_{v1}^{3}} \end{array}$$

It has been reported that this model is effective for low Reynolds number cases, provided that the mesh resolution is super fine with a wall *Y*^+^ ≤ 1 [9, 10].

### 2.3 SST K-*ω*

The menter SST K-*ω* is a combination of the Wilcox K-*ω* and the standard K-*ε* model [11]. The standard K-*ε* is transformed to K-*ω* by substituting *ε* = K*ω*[8]. These two equations are blended in order to utilise the advantage of the near wall treatment associated with the Wilcox model. It captures the sub-viscous layer effects in the inner layer, along with the standard K-*ω* model, which captures the outer layer effects.

The equations below describe the SST K-*ω* model

$$\begin{array}{c} \frac{\partial }{\partial t}\left(\rho k\right)+\frac{\partial }{\partial x_{i}}\left(\rho ku_{i}\right)=\frac{\partial }{\partial x_{j}}\left(\Gamma_{k}\frac{\partial k}{\partial x_{j}}\right)+G_{k}-Y_{k}+S_{k} \end{array}$$

$$\begin{array}{c} \frac{\partial }{\partial t}\left(\rho \omega \right)+\frac{\partial }{\partial x_{j}}\left(\rho \omega u_{j}\right)=\frac{\partial }{\partial x_{j}}\left(\Gamma_{\omega }\frac{\partial \omega }{\partial x_{j}}\right)+G_{\omega }-Y_{\omega }+D_{\omega }+S_{\omega } \end{array}$$

G_*ω*_ and G_*k*_ represent the generation of turbulent kinetic energy and the specific dissipation rate. Diffusivity is given by Γ_*ω*_ and Γ_*k*_. Dissipation is given by Y_*ω*_ and Y_*k*_. The source terms are given by S_*k*_ and S_*ω*_. The extra cross diffusion term D_*ω*_ is the blending function for the standard K-*ε* model and standard K-*ω* model.

$$\begin{array}{c} D_{\omega }=2\left(1-F_{1}\right)\rho \sigma_{\omega },2\frac{1}{\omega }\frac{\partial k}{\partial x_{j}}\frac{\partial \omega }{\partial x_{j}} \end{array}$$

### 2.4 Intermittency (*γ*) SST

Intermittency SST utilises the two equations of SST K-*ω* along with a third equation to *γ*[4].

$$\begin{array}{c} \frac{\partial \left(\rho \gamma \right)}{\partial t}+\frac{\partial \rho U_{j}\gamma }{\partial x_{j}}=P_{\gamma }-E_{\gamma }+\frac{\partial }{\partial x_{j}}\left[\left(\mu +\frac{\mu_{t}}{\sigma_{\gamma }}\right)\frac{\partial \gamma }{\partial x_{j}}\right] \end{array}$$

$$\begin{array}{c} \gamma =\frac{t_{turb}}{t_{lam}+t_{turb}} \end{array}$$

The Transition from laminar to turbulent is triggered using the correlation

$$\begin{array}{c} Re_{\theta c\left(T_{U},\lambda_{\theta }\right)}=C_{TU1}+C_{TU2}exp\left[-C_{TU3}T_{U}F_{PG}\left(\lambda_{\theta }\right)\right] \end{array}$$

### 2.5 K-kl-*ω*

K-kl-*ω* is developed for transition flows [12]. It accurately predicts the transition onset characteristic of the boundary layer. In this turbulence model, three equations are modeled for turbulent viscosity.

$$\begin{array}{c} \frac{Dk_{T}}{D}=P_{K_{T}}+R+R_{NAT}-\omega k_{T}-D_{T}\frac{\partial }{\partial x_{j}}\left[\left(v+\frac{\alpha_{T}}{\alpha_{k}}\right)\frac{\partial k_{T}}{\partial x_{j}}\right] \end{array}$$

$$\begin{array}{c} \frac{Dk_{L}}{D_{t}}=Pk_{L}-R-R_{NAT}-D_{L}+\frac{\partial }{\partial x_{j}}\left[v\frac{\partial k_{L}}{\partial x_{j}}\right] \end{array}$$

$$\begin{array}{c} \frac{D_{\omega }}{D_{t}}=C_{\omega 1}\frac{\omega }{k_{T}}P_{K_{T}}+\left(\frac{C_{\omega R}}{f_{W}}-1\right)\frac{\omega }{k_{T}}\left(R+R_{NAT}\right)-C\omega_{2}\omega^{2}+C\omega_{3}f_{\omega }\alpha_{T}f_{W}^{2}\frac{\sqrt{k_{T}}}{d^{3}}+\frac{\partial }{\partial x_{j}}\left[\left(v+\frac{\alpha_{\gamma }}{\alpha_{\omega }}\right)\frac{\partial \omega }{\partial x_{j}}\right] \end{array}$$

K_*T*_ is used to model the turbulent kinetic energy. The K_*L*_ equation is used to model the laminar kinetic energy. In the transition region, the energy associated with the Tollmien-Schlichting instabilities are captured by the K_*L*_ equation. The Inverse time scale *ω* is modelled as *ϵ* = *Kω*. The inverse time scale has been shown to reduce the intermittency effect in the outer turbulent boundary layer. It captures the adverse pressure gradients more accurately.

### 2.6 Transition (*γ-Re*_*θ*_) SST

This model is developed for transition flows. It is a combination of SST K-*ω* additionally coupled with intermittency *γ* and transition onset Reynolds no. *Re*_*θ*_ is the critical Reynolds number where the intermittency starts Menter et al. [13]. Four transport equations are solved; the first two equations are similar to SST K-*ω*.

The equation for intermittency and transition momentum thickness Reynolds number Menter et al. [14] is given as follows:

$$\begin{array}{c} \frac{\partial \left(\rho \gamma \right)}{\partial t}+\frac{\partial \rho U_{j}\gamma }{\partial x_{j}}=P_{x}\gamma 1-E_{\gamma 1}+P_{\gamma 2}-E_{\gamma 2}+\frac{\partial }{\partial x_{j}}\left[\left(\mu +\frac{\mu_{t}}{\sigma_{\gamma }}\right)\frac{\partial \gamma }{\partial x_{j}}\right] \end{array}$$

$$\begin{array}{c} \frac{\partial \left(\rho R{\bar{e}}_{\theta t}\right)}{\partial t}+\frac{\partial \left(\rho U_{j}R{\bar{e}}_{\theta t}\right)}{\partial x_{j}}=P_{\theta t}+\frac{\partial }{\partial x_{j}}\left[\sigma_{\theta t}\left(\mu +\mu_{t}\right)\frac{\partial R{\bar{e}}_{\theta t}}{\partial x_{j}}\right] \end{array}$$

The coupling of transition model with SST K-*ω* is performed by modifying the K- equation.

$$\begin{array}{c} \frac{\partial }{\partial t}\left(\rho k\right)+\frac{\partial }{\partial x_{i}}\left(\rho ku_{i}\right)=\frac{\partial }{\partial x_{j}}\left(\Gamma_{k}\frac{\partial k}{\partial x_{j}}\right)+G_{k}^{*}-Y_{k}^{*}+S_{k} \end{array}$$

$$\begin{array}{c} G_{k}^{*}=\gamma_{eff}{\bar{G}}_{k} \end{array}$$

$$\begin{array}{c} Y_{k}^{*}=min\left(max\left(\gamma_{eff},0.1\right),1.0\right)Y_{k} \end{array}$$

One of the major factor, which needs to be taken care of, in order to capture the transition behaviour and allow the above transition models to work, is to model the wall *Y*^+^. In order to resolve the viscous sub layer, it is crucial for the grid to be modelled with *Y*^+^ ≤ 1.

## 3 Numerical Analysis

**Fig 1:**
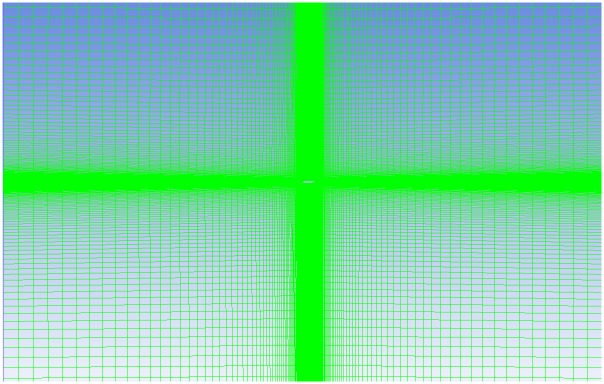
Domain with Structured Mesh.

The turbulence model testing on NACA 4415 airfoil is accomplished using the experimental data from Karthikeyan et al.[6]. 2D airfoil points are imported into Catia and a surface is generated. This Model is later imported into the ICEM module available in the Ansys package for meshing. A fluid domain is created around the airfoil as shown in Fig 1.

### 3.1 Domain Details

**Fig 2:**
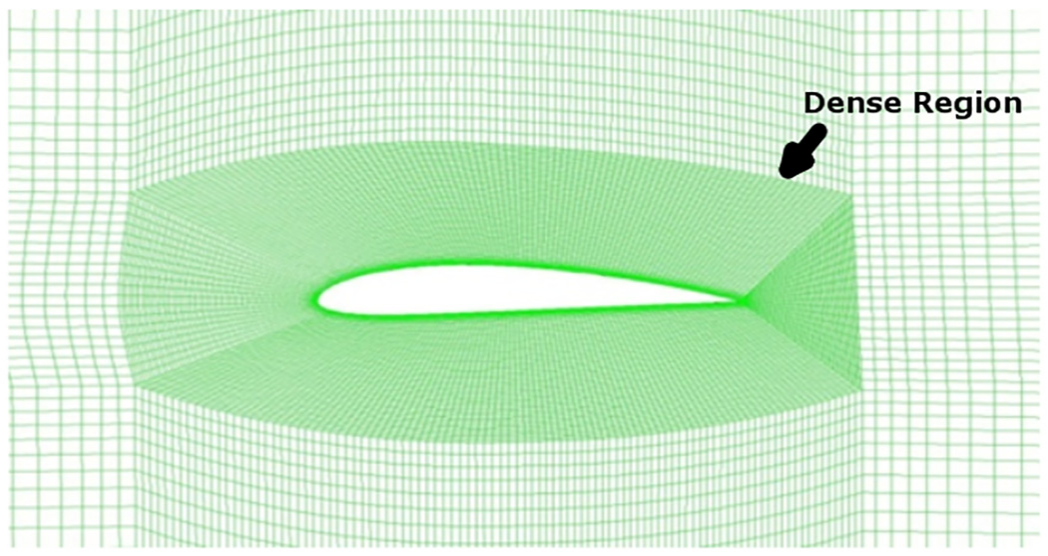
Structured mesh around the airfoil.

A rectangular domain, as shown in Fig 1, is created around the airfoil of unit chord length c. The inlet is kept at a distance of 20c from the airfoil and the outlet at 20c. The domain is extended 20c above and below the airfoil to avoid confinement effects. Meshing is carried out using ICEM. A blocking approach is used to discretize the domain into various zones. The zone closer to the airfoil has high grid density, obtained by enclosing a layer of very fine mesh Fig 2. In The outer zone the mesh density is gradually increased, thus the mesh density is coarsened as it goes outward away from the surface of the airfoil. A quad mesh is generated as shown in Fig 1. The wall *Y*^+^ is calculated, and the estimated distance is fixed at *Y*^+^ ≤ 1. It is important to take care of *Y*^+^ in order to capture the formation of the separation bubble.

### 3.2 Boundary Conditions

The input parameters such as pressure, density and viscosity are considered at sea level conditions. The input velocity at the inlet is set for a chord based Reynolds number of 120,000 Karthikeyan et al. [6]. The front, top and bottom walls of the rectangular domain are assigned as the inlet and the outlet is located behind the trailing edge of airfoil.

The turbulence intensity is set at 0.2% which is slightly above than the experimental reported value of 0.15% by Karthikeyan et al. [6]. The length scale was set at 2 and was not altered. It has been reported Butler et al. [15], and Cao [16] that the variation of the length scale has no effect whatsoever. They reported that the transition process and the turbulent structures are predominantly dependent on the turbulent intensity.

The flow is incompressible, hence a pressure based solver is used. SIMPLE pressure velocity coupling is implemented. The five turbulence schemes implemented are, S-A, SST K-*ω*, *γ*-SST, k-kl-*ω* and *γ-Re*_*θ*_ SST. The main purpose of implement these turbulence models is to check which is most effective to capture the flow behaviour. In order to avoid calculation errors, double precision is set. Second order discretization is set for pressure, momentum and other parameters. In order to maintain accuracy the convergence criterion is set at 1 × 10^−6^. The Angle of Attack (AoA) in the study by Karthikeyan et al. [6] is set at 6° and 18° and we have followed the same.

### 3.3 Grid Independence Check

The grid independence study was carried out, varying the number of nodes in the central region as shown in Fig 2. The grid was varied considering 100, 200, 300 and 400 nodes respectively in the central zone near to the airfoil. The *C*_*d*_ was set as the criterion for mesh dependency. The initial 100 nodes and 200 nodes grid was quite enough to capture the results. The 300-node mesh finally provided an accurate solution, and the comparison of *C*_*d*_ is shown in Table 1. This method increased the grid size but ensured that it was suitable for other turbulence models. The 300 nodes in the central region grid corresponds to 223k in the overall quad elements. Later, the simulation considering other turbulence models for the 223k grid was carried out.

**Table 1:** *C*_*d*_ comparison.

No of Nodes	*C*_*d*_ at 6° AoA S-A turbulence model
100	0.026549
200	0.026418
300	0.024658
400	0.024658

As the main aim was to model the separation bubble, the mesh size was increased, until further increases in the mesh yielded no difference in the *C*_*d*_ values. The advantage of using S-A model is that, the results are faster compared to other turbulence model. This is quite understandable as it uses only one transport equation to model the kinematic eddy viscosity. In the current simulation, the curvature correction is activated in Ansys Fluent in order to accurately capture the eddies around the curvature of the airfoil. The result showed that the values of lift of drag of S-A and *γ-Re*_*θ*_ SST were a match for the mesh selected. On further analysis of by plotting the BL plots and the contour plots the flow physics differed.

## 4 Results and Discussion

In this section, detail analysis of the experimental and numerical results are discussed for 6° and 18° AoA. The separation bubble capture and prediction of the various turbulence models is also compared. The flow physics has been explained by the co-efficient of pressure plots, boundary layer profiles, velocity contours, streamline profiles and the skin friction co-efficient plot.

### 4.1 6° AoA

The accuracy of the turbulence models in capturing the flow phenomenon is compared with the experimental results [6] at 6°. The initial comparison of the experimental results with XFLR5 (XFoil) has also been reported. A trivial comparison of the *C*_*l*_ and *C*_*d*_ is reported in Table 2.

**Table 2:** *C*_*l*_ and *C*_*d*_ comparison.

Turbulence model	*C*_*l*_	*C*_*d*_
XFoil	1.09	0.022
S-A	0.894	0.024658
SST K-*ω*	0.813	0.0288
*γ*-SST	0.789	0.0197
K-kl-*ω*	0.956	0.0327
*γ*-Re_*θ*_ SST	0.894	0.0247

The XFoil values reported are very high whereas the other turbulence models returned low values. Table 2 shows the value for 300 node mesh in the dense region Fig 2. It is quite clear that S-A and *γ*-Re_*θ*_ SST values are a close and the mesh is quite adequate for the current case.

### 4.2 Co-efficient of pressure *C*_*p*_ plots

**Fig 3:**
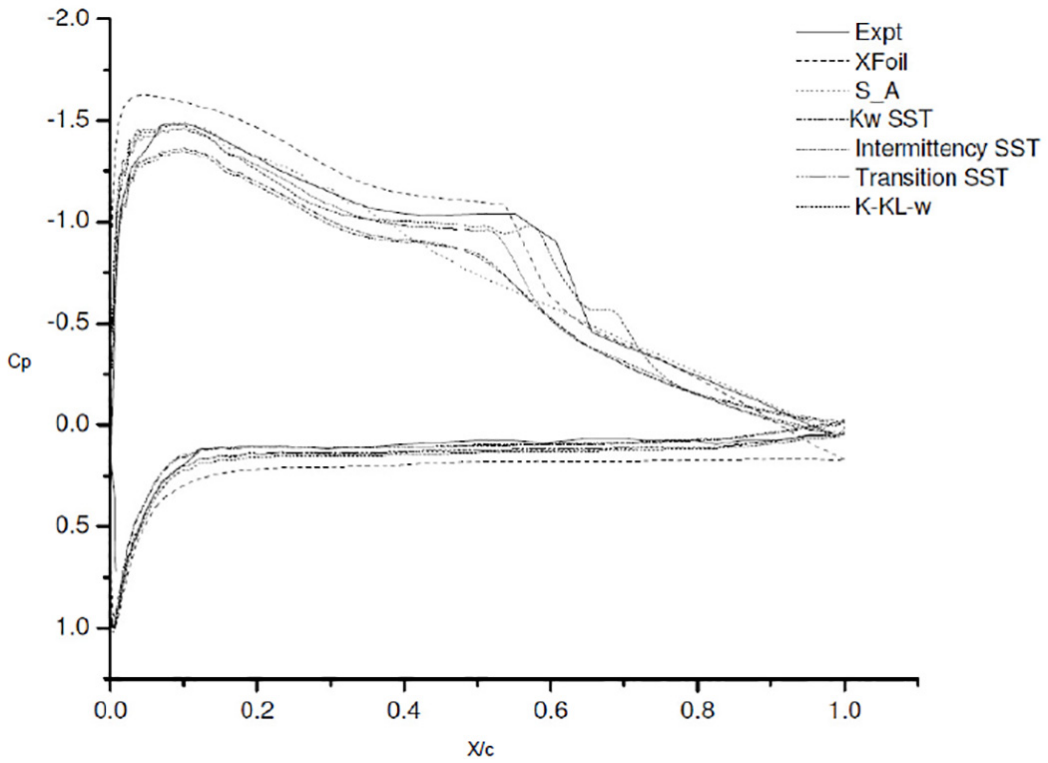
Co-efficient of pressure plot at 6° AoA.

The coefficient of pressure plots at 6° AoA is as shown in Fig 3 [data is provided in S1 File]. The comparative *C*_*p*_ plot with the experimental study of Karthikeyan et al. [6] is shown in Fig 3. The XFoil over predicts the lift and drag at 6° AoA, the separation and reattachment is predicted quite accurately.

As reported in literature S-A is designed for low Reynolds number and aerodynamic applications [3][5]. S-A does gives us a good approximation of lift and drag but not the flow physics. The convergence is quicker and the computation time required is much less. This might be due to the model’s inability to capture the changes in the length scale, due to separation from wall-bounded flows to free shear flow. The co-efficient of pressure plots signify a much better comparison of experimental and numerical result. A clear picture is obtained as these reveal a different story.

The *C*_*p*_ plot for SST K-*ω* Fig 3. shows a slight bump at 0.4c but it vanishes instantly. The formation of separation bubble and other instabilities that arise in the low Reynolds number flow, are not captured even with refined grid. Thus, it can be concluded SST K-*ω* is more accurate for fully turbulent flow as reported but not quite suitable for transition modeling. Similar to S-A, SST K-*ω* also gave faster convergence and utilized less computation time, but the results were quite similar to S-A. From the above comparison, it can be inferred that both S-A and SST K-*ω* are good turbulence models, very useful in case of flows where in the laminar separation bubble does not exist.

Next, we moved to other turbulence models, currently available to capture the transition effects namely, *γ*-SST, k-kl-*ω* and *γ*-Re_*θ*_ SST. These models have been reported to have the ability to capture the transition effects [5, 9, 11–14].

The *C*_*p*_ plots for *γ*-SST and k-kl-*ω* show the presence of bubble but do not capture the flow effectively. *γ*-Re_*θ*_ SST was most reliable in our case as it captured the initial laminar separation quite accurately at 0.35c, but under predicted the reattachment at approximately 0.6c. The Experimental results show that the laminar separation bubble for 6° AoA forms around 0.35c and extends till 0.65c. The turbulent reattachment takes place at 0.65c, whereby flow reattaches to the surface again. Choudary et al, [5] noticed this earlier reattachment of separation bubble in regard with *γ*-Re_*θ*_ SST and attributed it to the turbulence generated due to empirical formulation Menter et al. [14].

**Fig 4:**
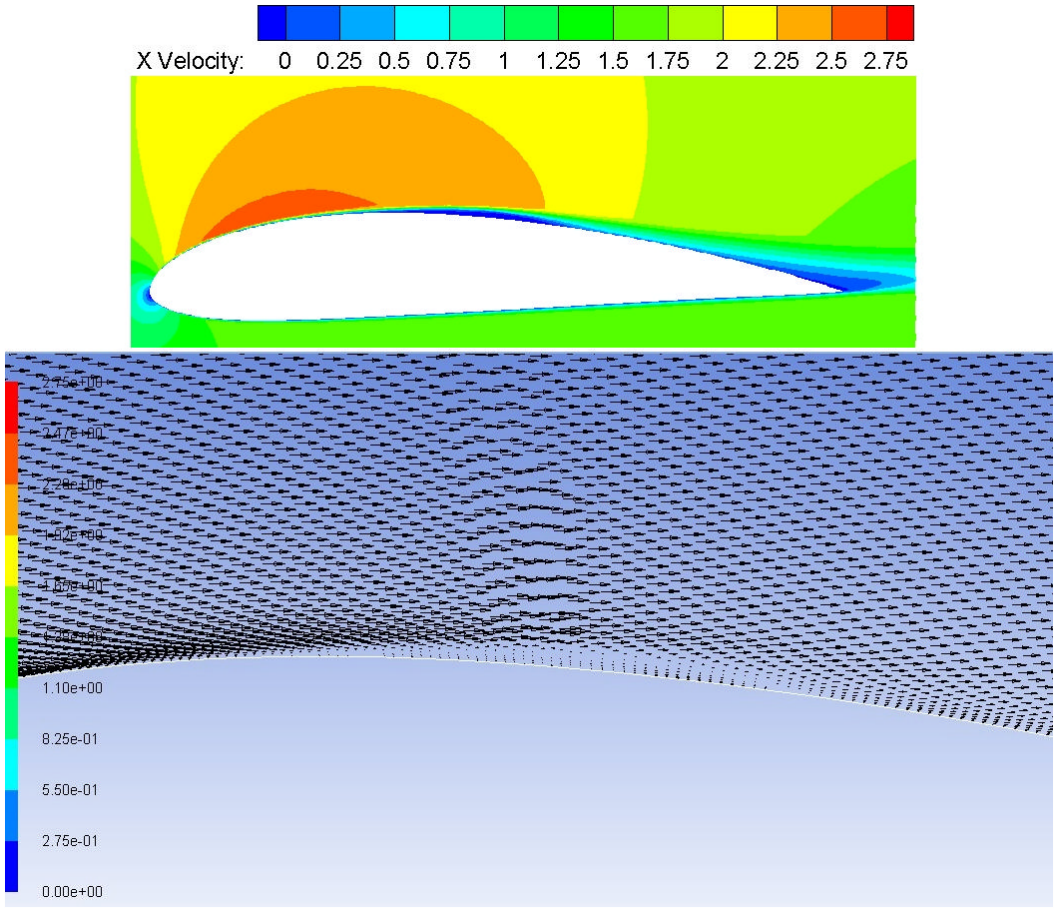
velocity contours and vectors along the top surface.

**Fig 5:**
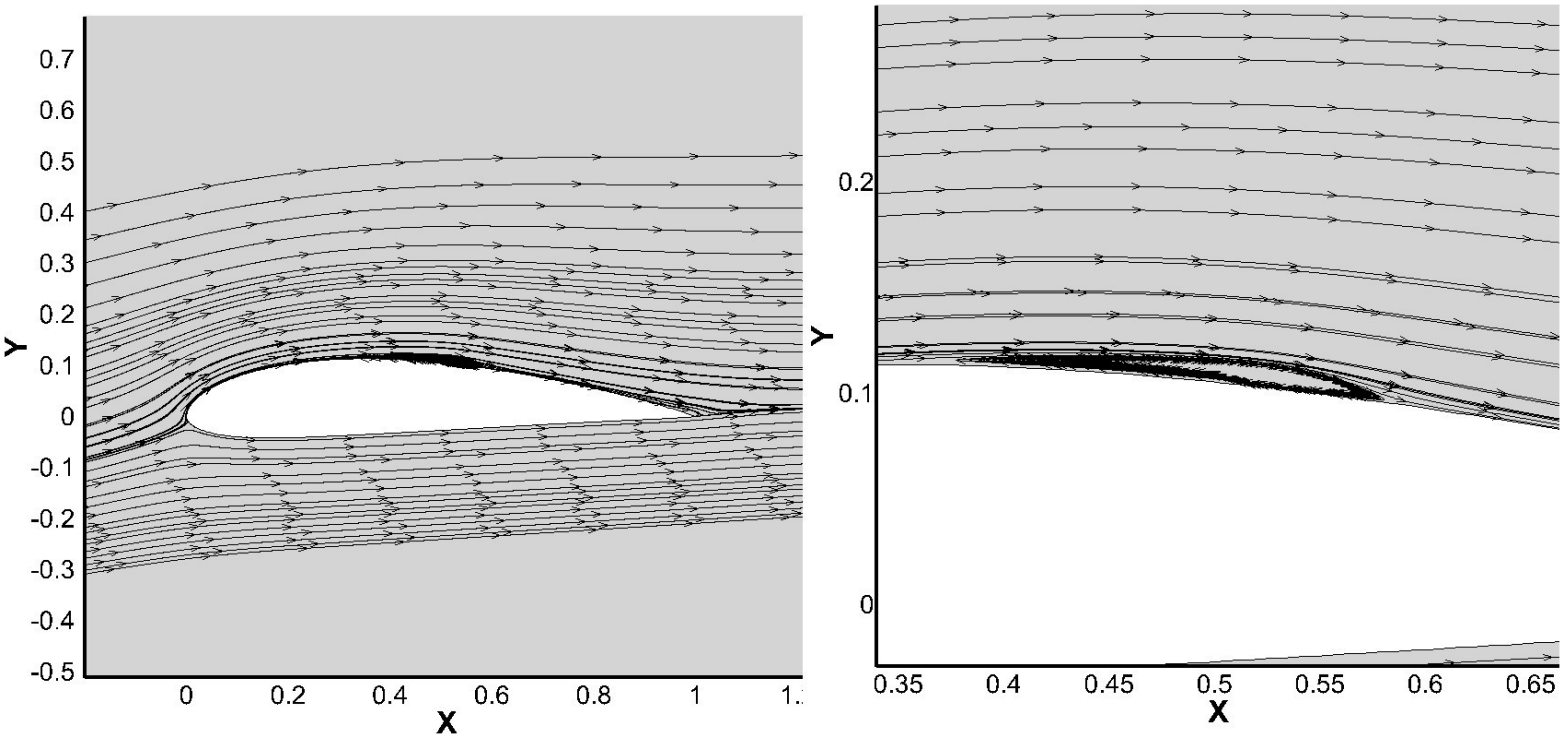
Streamlines along the airfoil at 6° AoA.

Fig 4 the contour plot and the vector plot show the transition onset, followed by the dead flow region in the core of the bubble. The turbulent reattachment is also clearly visualized, followed by redeveloping turbulent boundary layer. This behavior is not predicted and captured by S-A, SST K-*ω* *γ*-SST and k-kl-*ω* turbulence models. Furthermore the stream line plot clearly shows the bubble size matching with the experimental study Fig 5. The recirculation zone is also present in the core region which is the characteristic feature of the bubble. The *γ*-Re_*θ*_ SST model slightly under predicts the reattachment.

### 4.3 Boundary layer (BL) Plots

Further detailed analysis is carried out by plotting the boundary layer profiles on the upper surface of the airfoil. The BL plots are plotted with respect to each of the turbulence models.

**Fig 6:**
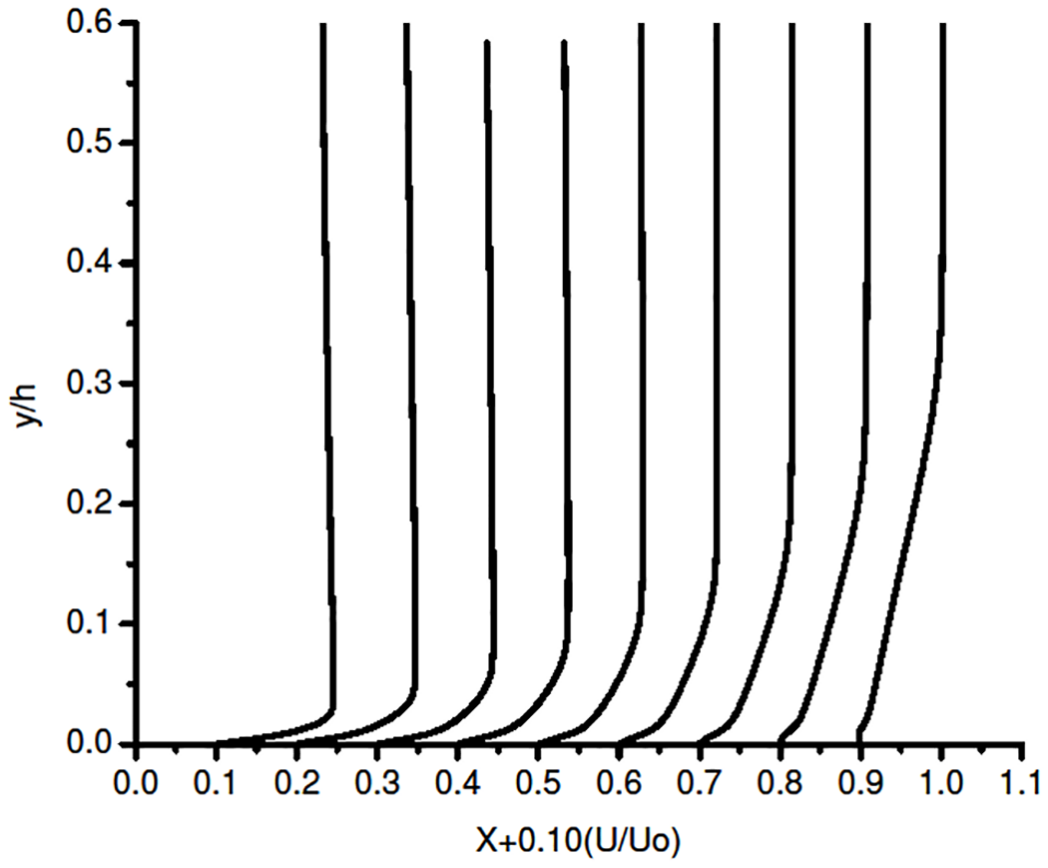
Boundary layer profile on the suction side S-A.

Fig 6 represent the boundary layer profile for S-A model at 6° AoA. The plot clearly shows that the flow is attached throughout the upper surface. The turbulence model fails to notice the separation and reattachment process. But it accurately captures the turbulent separation at the trailing edge of the airfoil.

**Fig 7:**
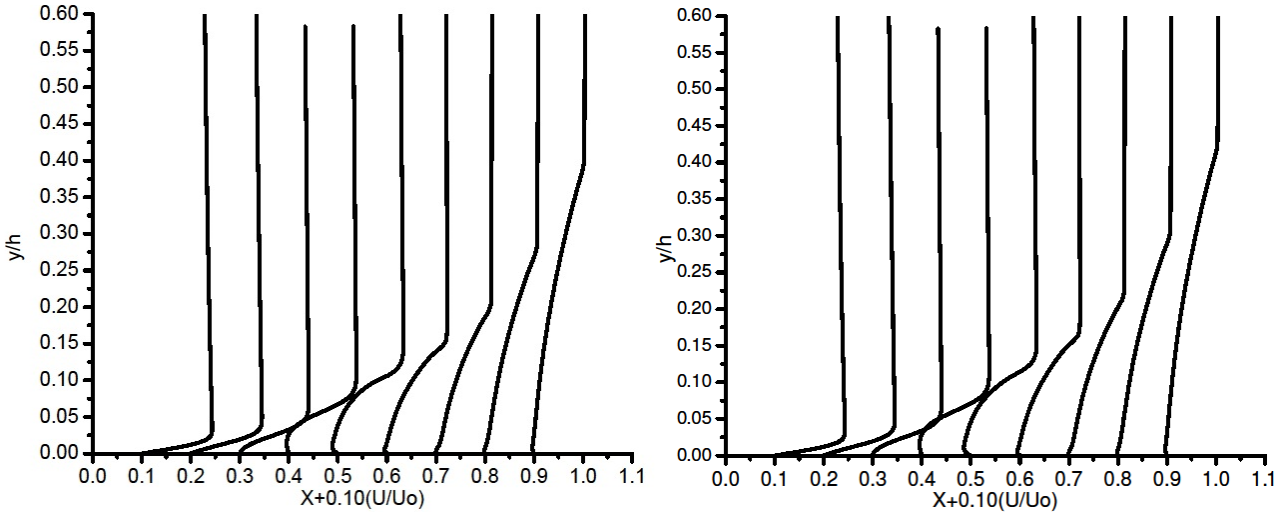
Boundary layer profile on the suction side (a) SST K-*ω* and (b) *γ*- SST.

Fig 7(a) does show signs of separation. The bubble is unstable and not accurately captured by SST K-*ω*. This behaviour has been noticed in the *C*_*p*_ plot too Fig 4.

The *γ*-SST model is an in-house developmental work of Ansys and most of the literature related to this is proprietary. An extra equation of the intermittency equation is activated, to enable this transition model. An extension is provided in Ansys Fluent under the SST turbulence model.

Fig 7(b) shows the BL plots for 3 equation *γ*-SST. The BL plot trend is similar to that observed in SST K-*ω*. The separation bubble forms and vanishes quite rapidly. The BL plots show identical behavior; the initial laminar separation is detected at at 0.4c but the reattachment is not predicted. It directly enters into the turbulent separation.

**Fig 8:**
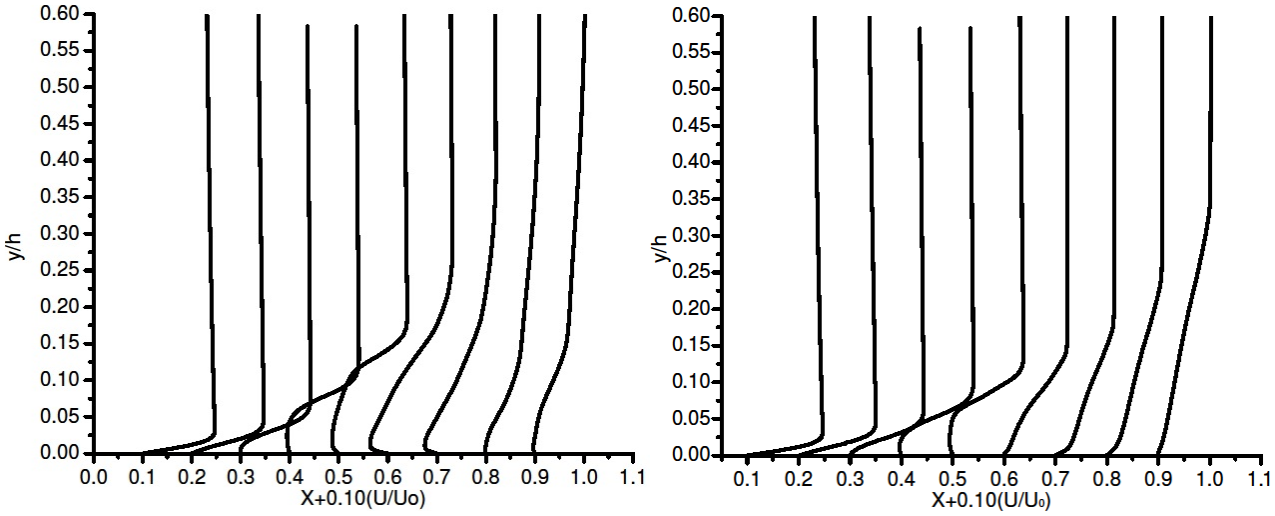
Boundary layer profile on the suction side (a) k-kl-*ω* and (b) *γ*-Re_*θ*_ SST.

Fig 8(a) shows the BL plot using k-kl-*ω* model. It showed erratic behavior initially, further altering the under-relaxation factors, the simulation for 6° AoA took more than a week to obtain a converged solution. This model requires huge computational resources. The model did not accurately capture the separation bubble in the present case. The pressure co-efficient plot Fig 4 and boundary layer plot Fig 8(a) clearly demonstrated the separation bubble prediction capability of k-kl-*ω*.

Fig 8(b) shows the BL plot for *γ*-Re_*θ*_ SST model. As reported in an earlier section uses four transport equations. Two are taken from SST K-*ω*, the other two include intermittency and the transition onset Reynolds number equation Re_*θ*_. In the current case, this model showed very good results, accurately predicting the transition onset, laminar seperation bubble formation and the turbulent reattachment Fig 8(b). These flow features were not noticed in any of the turbulence models except k-kl-*ω*, which did model the separation bubble to a certain extent.

The BL profile plot in Fig 8(b) along the airfoil, shows the flow on the suction side. The flow is attached till 0.3c. The plots show transition in the flow at 0.35c. This transition gives rise to the formation of the separation bubble. At 0.5c, the flow is completely detached from the surface, which shows total separation. The partial reattachment starts at 0.6c. Reattached flow can be noticed at 0.65c. Thus *γ*-Re_*θ*_ SST model prediction capability concurs with the experimental study of Karthikeyan et al. [6] [Figs 6, 7 (a) (b) and 8 (a) (b) data is provided in S2 File].

### 4.4 Skin Friction co-efficient

**Fig 9:**
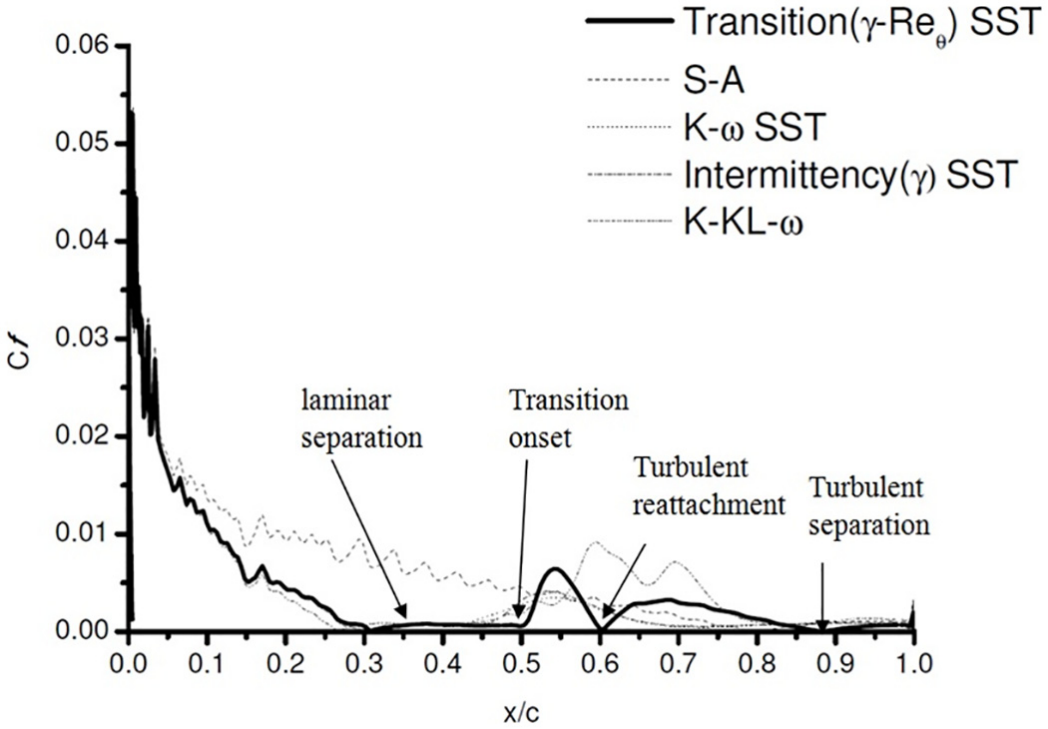
Skin friction coefficient on the pressure side of the airfoil.

The skin friction plot Fig 9 [data is provided in S3 File]. clearly highlights the behavior, indicating that four of the five turbulence models applied are not suitable for our case. The authors have previous carried out extensive analysis using S-A turbulence models at low Reynolds number [17]. The phenomenon reported in this work was not reported or noticed for S-A simulations. Thus, it shows the importance of using accurate turbulence model. For the current case *γ*-Re_*θ*_ SST shows the capability of predicting the separation and the extension of bubble size accurately (Figs 4 and 5).

The skin friction coefficient plot shows the accuracy of Transition *γ*-Re_*θ*_ SST. The profile accurately predicts the initial separation, the separated region and the reattachment. In comparision with the *C*_*p*_ plots, which show that the k-kl-*ω* also predicts the separation. Fig 9 shows quite clearly that even though k-kl-*ω* predicts the separation, it does not accurately model the reattachment at 6° AoA for the present case. Skipping the turbulent reattachment, it moves directly into the turbulent separation region. This might be due to the reduced intermittency, predicting non accurate reattachment.

### 4.5 18° AoA

**Fig 10:**
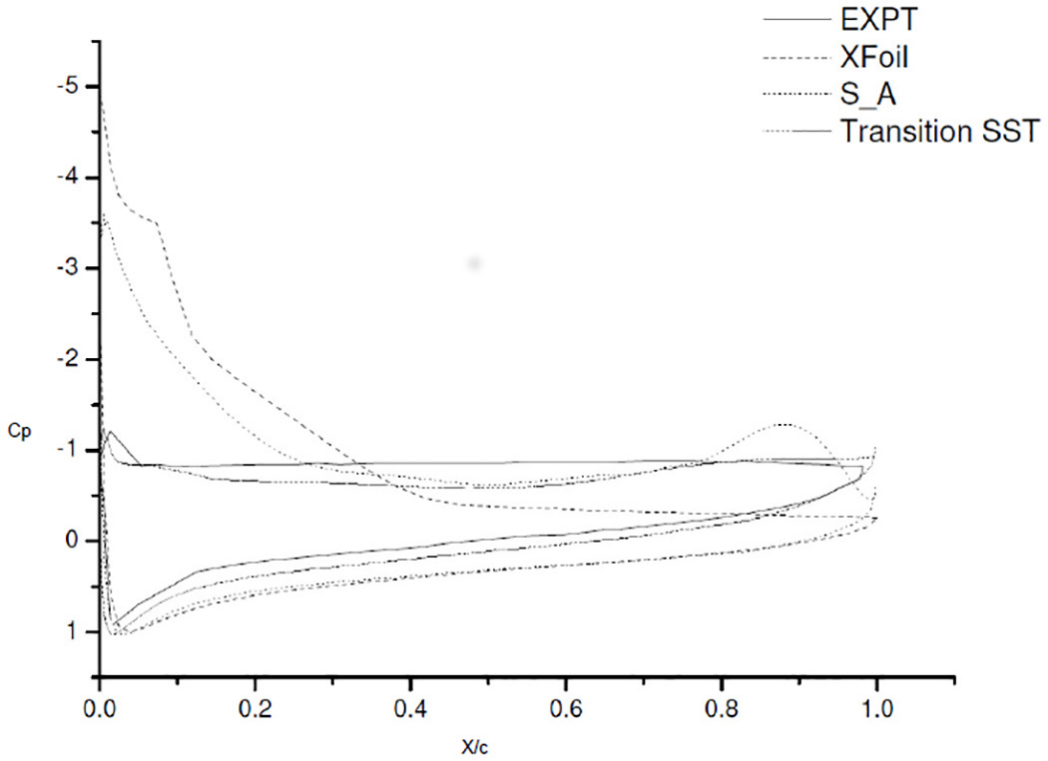
Co-efficient of pressure plot at 18° AOA.

The results in Fig 10 show that the right choice was made. The flow over the airfoil surface at 18° AoA is completely separated. The airfoil experiences total flow separation at this AoA. The *C*_*p*_ plot of *γ*-Re_*θ*_ SST and the experimental study of Karthikeyan et al. [6] show this behaviour and are close enough. The XFoil results are exaggerated, followed by the S-A turbulence model. This clearly indicates that simply comparing the lift and drag forces may be misleading. Pressure contours give a clearer picture of the flow physics and the forces acting over the airfoil surface.

The work was mostly related to predicting separation bubble formation and its travel in the span-wise direction. The flow is completely separated at high AoA, Thus only S-A and *γ*-Re_*θ*_ SST were compared. The main reason for rejecting k-kl-*ω* in this case was the instability in computation and the convergence issues faced during the simulation of the flow at 6° and 18° AoA.

### 4.6 Simulation Time

In this analysis, it was found that the S-A took the least amount of time for the simulation to obtain a converged solution. k-kl-*ω* was the most time consuming and computationally very expensive, for the available computational resources. At 18° AoA k-kl-*ω* did not return a stable solution for the unsteady simulation as well. Tweaking the under-relaxation parameters too did not yield any substantial results.

**Fig 11:**
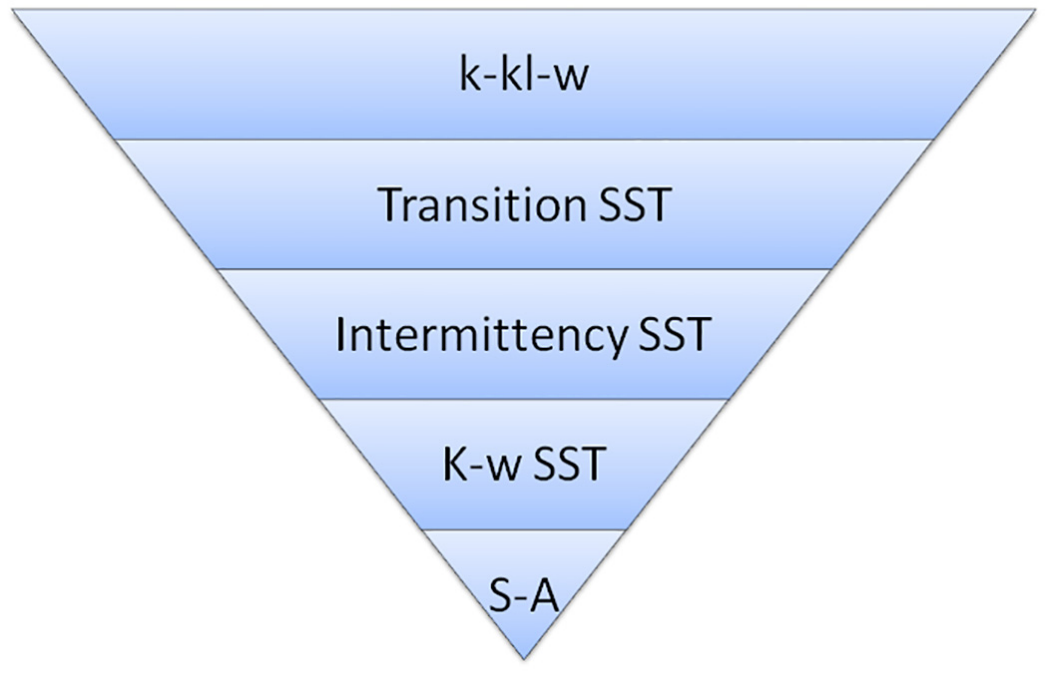
Computational time requirement.

The Fig 11 shows the Computational time required by each of the turbulence models, in order to obtain a converged solution for the current simulation. For S-A the solution is obtained in less than half an hour. k-kl-*ω* had to be run for around 200,000 iterations, to obtain a converged solution, and required more than a week on 8 core processor with 24GB of RAM.

**Fig 12:**
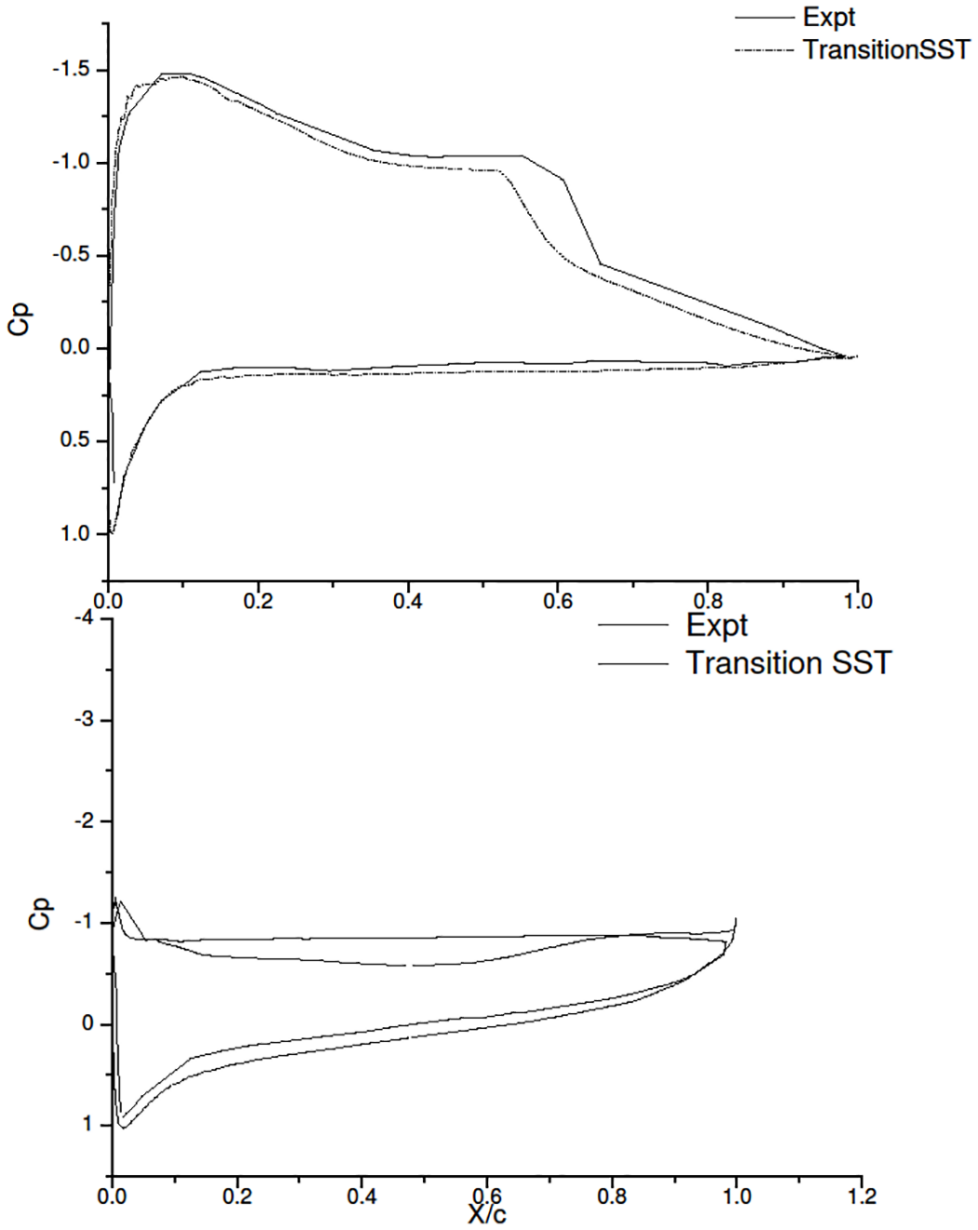
*C*_*p*_ Expt and *γ*-Re_*θ*_ SST.

Fig 12 shows that *γ*-Re_*θ*_ SST turbulence model is a good choice to model the flow behavior for low Reynolds number in this case. The *C*_*p*_ plots shows the pressure distribution at 6° and 18° AoA. Results of *γ*-Re_*θ*_ SST are comparable with the experimental study Karthikeyan et al. [6].

## 5 Conclusion

The following conclusions have been drawn from the current CFD study, which was carried out on NACA4415 airfoil at Reynolds no 120,000.

1. S-A is a robust turbulence model and can provide a very good initial guess for low Reynolds number aerodynamic flows. The *C*_*l*_ and *C*_*d*_ results clearly suggest that the results of S-A are in agreement with those obtained from *γ* — *Re*_*θ*_ SST at 6° AoA.
2. For the current simulation, the K-*ω* SST and Intermittency K-*ω* SST provide identical results, with slight changes in lift and drag values. Both the models do show a slight formation of the separation bubble, but fail to capture it. K-*ω* SST has been known to provide good results for external aerodynamic cases when the flow is fully turbulent. From the results, it can inferred that K-*ω* SST and intermittency SST are clearly not suitable for the current case.
3. The k-kl-*ω* also gave very good results at low AoA (6°). The major reason for the rejection of this turbulence model was the computational time and the resources that it required. The *C*_*p*_ plots showed that the model did provide results closer to the experimental results. However the skin friction results showed the true behaviour, thus this model cannot be used for the current case.
4. For the current case, only *γ-Re*_*θ*_ SST provides reliable results, compared to other turbulence models. The model accurately captures the flow physics in the low AoA (6°), as well as in the high AoA (18°) case.
  The results obtained show the values of the experimental study and the current CFD study are found to be in good agreement. This Study clearly shows that capturing the transition behaviour, for low Reynolds numbers flows, needs an accurate turbulence model. In the present case, *γ-Re*_*θ*_ SST is preferred model as it predicts the flow behaviour both at low and high AoA, accurately and in a short duration of time.

## Supporting Information

S1 File Links to online data files for Cp plots [Fig 3]. (RAR)

S2 File Links to online data files for Boundary layer plots [Figs 6, 7 (a) (b) and 8 (a) (b)]. (RAR)

S3 File Links to online data files for Skin friction plots [Fig 9]. (RAR)
